# Chest compression rates of 60/min versus 90/min during neonatal cardiopulmonary resuscitation: a randomized controlled animal trial

**DOI:** 10.3389/fped.2023.1214513

**Published:** 2023-08-16

**Authors:** Marlies Bruckner, Megan O'Reilly, Tze-Fun Lee, Po-Yin Cheung, Georg M. Schmölzer

**Affiliations:** ^1^ Centre for the Studies of Asphyxia and Resuscitation, Neonatal Research Unit, Royal Alexandra Hospital, Edmonton, AB, Canada; ^2^ Division of Neonatology, Department of Pediatrics and Adolescent Medicine, Medical University of Graz, Graz, Austria; ^3^ Department of Pediatrics, Faculty of Medicine and Dentistry, University of Alberta, Edmonton, AB, Canada

**Keywords:** infant, newborn, chest compression, resuscitation, rate, sustained inflation, asphyxia, cardiac arrest

## Abstract

**Background:**

To compare chest compression (CC) rates of 60/min with 90/min and their effect on the time to return of spontaneous circulation (ROSC), survival, hemodynamic, and respiratory parameters. We hypothesized that asphyxiated newborn piglets that received CC at 60/min vs. 90/min during cardiopulmonary resuscitation would have a shorter time to ROSC.

**Methods:**

Newborn piglets (*n* = 7/group) were anesthetized, tracheotomized and intubated, instrumented and exposed to 45 min normocapnic hypoxia followed by asphyxia and cardiac arrest. Piglets were randomly allocated to a CC rate of 60/min or 90/min. CC was performed using an automated CC machine using CC superimposed with sustained inflation. Hemodynamic parameters, respiratory parameters, and applied compression force were continuously measured.

**Results:**

The mean (IQR) time to ROSC was 97 (65–149) s and 136 (88–395) s for CC rates of 60/min and 90/min, respectively (*p* = 0.31). The number of piglets that achieved ROSC was 5 (71%) and 5 (71%) with 60/min and 90/min CC rates, respectively (*p* = 1.00). Hemodynamic parameters (i.e., diastolic and mean blood pressure, carotid blood flow, stroke volume, end-diastolic volume, left ventricular contractile function) and respiratory parameters (i.e., minute ventilation, peak inflation and peak expiration flow) were all similar with a CC rate of 60/min compared to 90/min.

**Conclusion:**

Time to ROSC, hemodynamic, and respiratory parameters were not significantly different between CC rates of 60/min vs. 90/min. Different CC rates during neonatal resuscitation warrant further investigation.

## Introduction

A 3:1 compression-to-ventilation (C:V) ratio with 90 chest compressions (CC) and 30 inflations to achieve approximately 120 events per minute is the current consensus of science and treatment recommendations (CoSTR) for neonatal resuscitation (1, 2). However, the optimal CC rate to optimize coronary and cerebral perfusion during cardiopulmonary resuscitation (CPR) remains unknown.

Over the last decade, we have studied CC superimposed by sustained inflation (SI = CC + SI) (3). CC + SI achieves passive lung aeration thereby increasing minute ventilation and oxygenation. Further, the constant distending pressure results in increased intrathoracic pressure leading to significantly higher pulmonary and carotid blood flow (3). Schmölzer et al. compared CC + SI with a CC rate of 120/min with 3:1 C:V and reported a significantly reduced time to return of spontaneous circulation (ROSC) [38 (23–44) s vs. 143 (84–303) s, *p* = 0.0008] and improved survival [7/8 (87.5%) vs. 3/8 (37.5%), *p* = 0.038] in bradycardic piglets (4). Further piglet studies have compared CC + SI with a CC rate of 90/min vs. 120/min (5), CC + SI with a rate of 90/min vs. 3:1 C:V (6). CC + SI with a rate of 90/min also significantly reduced the median (IQR) time to ROSC compared to 3:1 C:V [34 (28–156) s vs. 210 (72–300) s, *p* = 0.048] (6). Comparison of different compression rates using CC + SI showed similar time to ROSC when using 90/min vs. 120/min (5), and 90/min vs. 180/min (7). However, no study has examined lower CC rates to assess if this would improve cardiac output. Therefore we aimed to examine CC + SI with rates of 60/min vs. 90/min and their effect on the time to ROSC, survival, and hemodynamic and respiratory parameters. We hypothesized that a CC rate of 60/min compared to 90/min during CPR would result in a shorter time to ROSC in asphyxiated newborn piglets.

## Materials and methods

**Figure 1:**
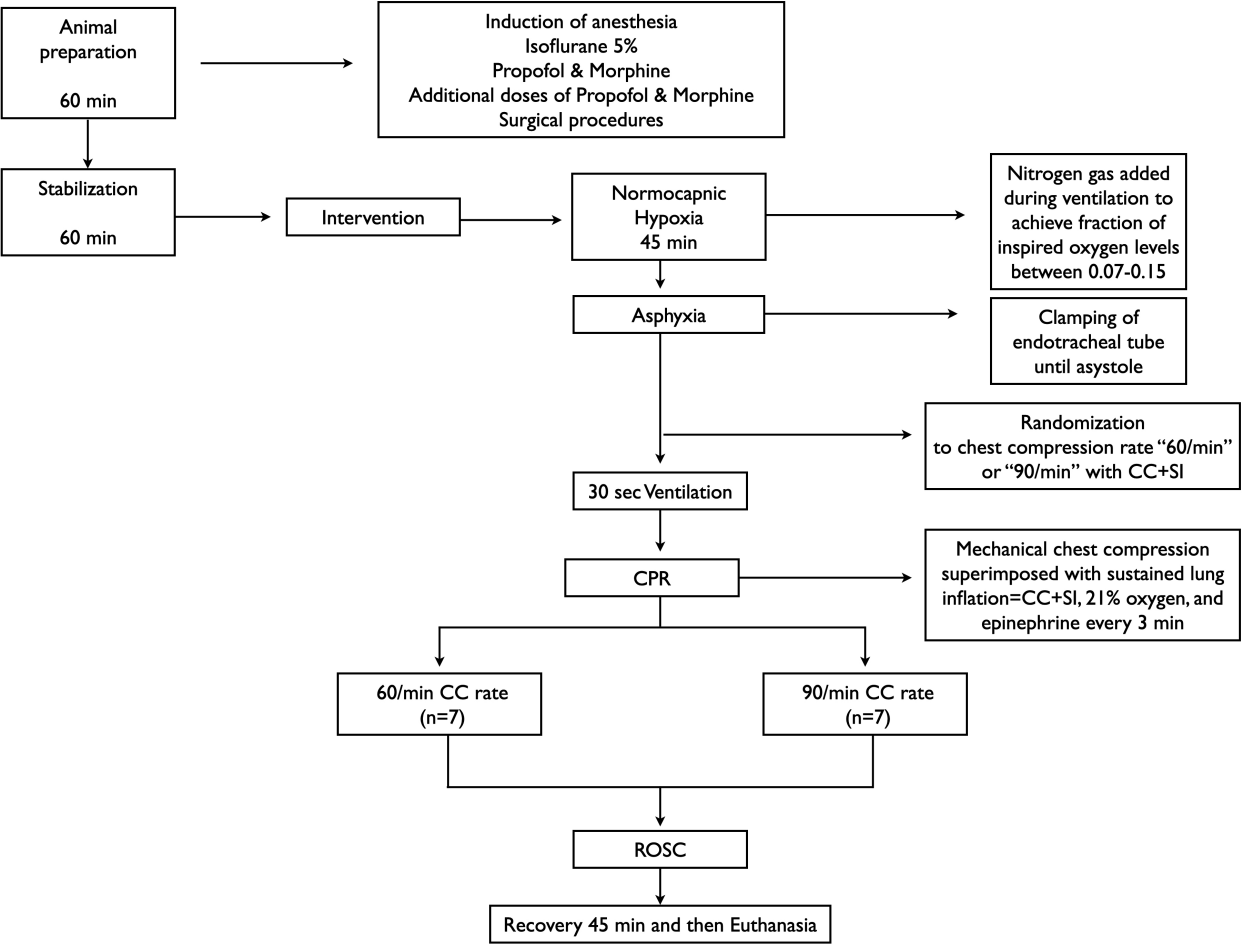
Study flow diagram.

All experiments were conducted between January and November 2020 in accordance with the guidelines and approval of the Animal Care and Use Committee (Health Sciences), University of Alberta (AUP00001764), presented according to the ARRIVE guidelines (8), and registered at preclincialtrials.eu (PTCE0000148). A graphical display of the study protocol is presented in Figure 1. The authors declare that all supporting data are available within the article.

### Randomization

Piglets were randomly allocated to CC with rate of 60/min or CC with rate of 90/min with a 1:1 randomization with variable block sizes using a computer-generated randomization program (http://www.randomizer.org). Sequentially numbered, sealed, brown envelopes containing the group allocation were opened during the experiment (Figure 1).

### Sample size and power estimates

The primary outcome measure was the time of CPR to achieve ROSC. Our previous studies showed a mean (standard deviation, SD) time to achieve ROSC of 220 (25) s with a CC rate of 90/min. We hypothesized that a CC rate of 60/min would reduce the time to achieve ROSC. A sample size of 7 per group would be sufficient to detect a clinically important (20%) reduction in time to ROSC (i.e., 176 s vs. 220 s) with 90% power and a 2-tailed alpha error of 0.05.

### Blinding

One investigator (TFL) opened the randomization envelope and entered the CC rate on the automated CC machine settings. GMS assessed cardiac arrest (confirmed asystole) and was blinded to group allocation until mechanical CC commenced. All other group members were also blinded to group allocation prior to commencement of mechanical CC. The statistical analysis was blinded to group allocation, and the data was unblinded following completion of the analysis.

### Inclusion and exclusion criteria

Newborn mixed-breed piglets (0–3 days of age) obtained on the day of experimentation from the University Swine Research Technology Center were included. There were no exclusion criteria.

### Animal preparation

Piglets were instrumented as previously described with some modifications (4). Following the induction of anesthesia using isoflurane, piglets were intubated via tracheostomy, and mechanical ventilation (Sechrist infant ventilator model IV-100; Sechrist Industries, Anaheim, CA) was commenced at a respiratory rate of 20 breaths/min, peak inflation pressure of 25 cmH_2_O, and positive end-expiratory pressure of 5 cmH_2_O. Oxygen saturation was kept within 90–100%, glucose level and hydration were maintained with an intravenous infusion of 5% dextrose at 10 ml/kg/h. During the experiment, anesthesia was maintained with intravenous propofol 5–10 mg/kg/h and morphine 0.1 mg/kg/h. Additional doses of propofol (1–2 mg/kg) and morphine (0.05–0.1 mg/kg) were also given as needed, and their body temperature was maintained at 38.5–39.5°C by using an overhead warmer and a circulating water heating pad.

### Hemodynamic parameters

A 5-French Argyle® (Klein-Baker Medical Inc. San Antonio, TX) double-lumen catheter was inserted into the right femoral vein for fluid administration and medications. A 5-French Argyle® single-lumen catheter was inserted above the right renal artery via the femoral artery for continuous arterial blood pressure monitoring and arterial blood gas measurements. The right common carotid artery was exposed and encircled with a real-time ultrasonic flow probe (2 mm; Transonic Systems Inc., Ithica, NY) to measure carotid blood flow. A Millar® catheter (MPVS Ultra® ADInstruments, Houston, TX) was inserted into the left ventricle via the left common carotid artery for continuous measurement of stroke volume, end-diastolic volumes, dP/dt_max_ (maximal rate of rise of left ventricular pressure), and dP/dt_min_ (minimum rate of change of ventricular pressure), which served as a surrogate for cardiac output. Due to the size difference between the Millar catheter and left ventricle longitudinal axis, which poses a limitation for the accuracy of *in vivo* volume measurement, an alpha factor =0.46, based on comparison between Millar's recording and direct echocardiographic measurements in three piglets, was used to correct the conductance volume (9).

Piglets were placed in the supine position and allowed to recover from surgical instrumentation until baseline hemodynamic measures were stable (minimum of one hour). The ventilator rate was adjusted to keep the partial arterial CO_2_ between 35 and 45 mmHg as determined by periodic arterial blood gas analysis. Arterial blood pressure, heart rate, and percutaneous oxygen saturation were continuously measured and recorded throughout the experiment with a Hewlett Packard 78833B monitor (Hewlett Packard Co., Palo Alto, CA).

### Respiratory parameters

A respiratory function monitor (NM3, Respironics, Philips, Andover, MA) continuously measured tidal volume, airway pressures, gas flow, and end-tidal CO_2_. The sensor was placed between the endotracheal tube and the ventilation device. Tidal volume was calculated by integrating the flow signal, and end-tidal CO_2_ was measured using a nondispersive infrared absorption technique (10, 11). The accuracy for gas flow was ±0.125l/min, and the end-tidal CO_2_ was ±2 mmHg.

### Automated chest compression machine

The automated CC machine was custom designed in our laboratory. The settings used for the automated CC machine were anterior-posterior compression depth 33%, acceleration of compression 500 cm/s^2^, speed of recoil 50 cm/s, a simulated two-thumb technique, and a CC rate of 90/min or 60/min, according to group allocation (12–14).

### Force measurement

A FlexiForce A201 sensor (TekScan, Boston, MA) was placed on the bottom of the plunger of the automated CC machine to measure the applied compression force. The applied compression force was recorded with Arduino Software (Somervile, MA) with a sample rate of 200 Hz (12, 13).

### Experimental protocol

Post-transitional piglets were randomized into two groups: “CC rate 60/min” or “CC rate 90/min”. Following surgical instrumentation and stabilization, the piglets were placed onto the automated CC machine, which was placed on the surgical bed. The piglets' anterior-posterior chest diameter was measured from the sternum to the vertebrae touching the bed (anterior to posterior) with a measuring tape, and the anterior-posterior depth of 33% was calculated (12, 13). Piglets were then exposed to 45 min of normocapnic hypoxia, which was followed by asphyxia. Normocapnic hypoxia was achieved by reducing the fraction of inspired oxygen and adjusting the ventilation rate. Asphyxia was achieved by disconnecting the ventilator and clamping the endotracheal tube until asystole. Asystole was defined as zero carotid blood flow and no audible heartbeat during auscultation. Fifteen seconds after asystole, positive pressure ventilation was provided for 30 s with a Neopuff T-Piece (Fisher & Paykel, Auckland, New Zealand) with 21% oxygen, peak inspiratory pressure of 30 cmH_2_O, positive end-expiratory pressure of 5 cmH_2_O, gas flow of 8l/min, and at a rate of 50/min. After 30 s of positive pressure ventilation, mechanical CC using our automated CC machine was initiated (12, 13) using 21% oxygen (14, 15), with an anterior-posterior compression depth of 33% (12, 13), and continuous CC during sustained inflation (CC + SI) was delivered with a peak inspiratory pressure of 30 cmH_2_O for 30 s (16–18). Sustained inflations were provided for 30 s, then interrupted for 1 s before a further 30 s of sustained inflation was provided, this sequence was continued until ROSC (4). CC + SI were provided for a maximum time of 10 min, and if no ROSC was achieved resuscitation efforts were stopped. Epinephrine (0.02 mg/kg per dose) was administered intravenously 2 min after the start of positive pressure ventilation and thereafter every 3 min until ROSC with a maximum of three doses (1, 2), as the maximum resuscitation time was 10 min. The administration of epinephrine was immediately followed by a saline flush of 3 ml (19, 20). ROSC was defined as an unassisted heart rate >100/min for at least 15 s. After ROSC, piglets recovered for 45 min before being euthanized with an intravenous overdose of sodium pentobarbital (120 mg/kg). If there was no ROSC, piglets were euthanized immediately with an intravenous overdose of sodium pentobarbital (120 mg/kg). Autopsies were performed in all piglets to assess for injuries to the sternum, ribs, heart or lungs (i.e., bruising, abrasions, contusions, fractures).

### Data collection and statistical analysis

The demographics of study piglets were recorded. Transonic flow probe, heart rate and pressure transducer outputs were digitized and recorded with LabChart® programming software (AD Instruments, Houston, TX). Airway pressures, gas flow, tidal volume, and end-tidal CO_2_ were measured and analyzed using Flow Tool Physiologic Waveform Viewer (Philips Healthcare, Wallingford, CT, USA). For all respiratory parameters, the median values for each piglet during CPR were calculated first, and then the mean of the median was calculated for comparison.

The data are presented as the mean (standard deviation—SD) for normally distributed continuous variables and median (interquartile range—IQR) when the distribution was skewed. The data were tested for normality (Shapiro–Wilk and Kolmogorov–Smirnov test) and compared using t tests or rank sum for normally or skewed distributed data. *P*-values are 2-sided, and *p* < 0.05 was considered statistically significant. Statistical analyses were performed with SigmaPlot (Systat Software Inc, San Jose, USA).

## Results

Fourteen newborn mixed breed piglets (0–3 days, ranging in weight from 1.9–2.4 kg) were obtained on the day of the experiment and were randomly assigned to CC rate of 60/min (*n* = 7) or 90/min (*n* = 7). Parameters at baseline or at start of CPR (end of asphyxia) were not different between groups (Table 1). The median (IQR) duration of asphyxia was 440 (310–490) s and 440 (280–506) s in the 60/min and 90/min CC groups, respectively (*p* = 1.00).

**Table 1:** Characteristics of newborn piglets at baseline, commencement of resuscitation, and post-resuscitation.

	60/min CC rate (*n* = 7)	90/min CC rate (*n* = 7)	*p*-value
Baseline characteristics			
Age (days)	2 (1–3)	2 (1–3)	1.00
Weight (kg)	2.2 (1.9–2.4)	2.2 (2.0–2.4)	0.90
Sex (Male/Female)	6/1	2/5	0.10
Heart rate (bpm)	142 (132–183)	144 (142–159)	1.00
Mean arterial blood pressure (mmHg)	60 (53–64)	53 (52–61)	0.26
Diastolic pressure (mmHg)	44 (40–51)	40 (37–44)	0.11
Carotid blood flow (ml/min)	27 (22–42)	36 (34–51)	0.81
Cerebral oxygenation (%)	35 (33–45)	32 (32–41)	0.13
Arterial pH	7.52 (7.49–7.62)	7.47 (7.46–7.51)	0.11
PaO_2_ (torr)	71 (60–86)	62 (61–74)	0.54
PaCO_2_ (torr)	34.4 (29.6–37.5)	36.3 (35.0–38.9)	0.26
Base excess (mmol/L)	3 (1–4)	3 (2–4)	1.00
Lactate (mmol/L)	3.3 (2.3–3.6)	3.6 (2.5–4.2)	0.62
Characteristics at commencement of CPR (end of asphyxia)			
Heart rate (bpm)	0 (0–0)	0 (0–0)	1.00
Mean arterial blood pressure (mmHg)	0 (0–0)	0 (0–0)	1.00
Diastolic pressure (mmHg)	0 (0–0)	0 (0–0)	1.00
Carotid blood flow (ml/min)	0 (0–0)	0 (0–0)	1.00
Arterial pH	6.58 (6.50–6.75)	6.58 (6.54–6.68)	0.95
PaO_2_ (torr)	13 (10–16)	14 (13–20)	0.30
PaCO_2_ (torr)	85 (82–112)	102 (67–121)	0.84
Base excess (mmol/L)	−30 (−30–−19)	−29 (−30–−26)	0.95
Lactate (mmol/L)	17 (16–19)	16 (16–19)	0.63
Characteristics immediately after ROSC			
Heart rate (bpm)	205 (172–230)	180 (165–212)	0.31
Mean arterial blood pressure (mmHg)	71 (50–87)	56 (43–75)	0.55
Diastolic pressure (mmHg)	48 (37–73)	43 (32–60)	0.55
Carotid blood flow (ml/min)	20 (19–52)	23 (21–30)	0.83
Arterial pH	6.87 (6.72–6.93)	6.78 (6.52–6.84)	0.55
PaO_2_ (torr)	68 (43–81)	77 (40–109)	1.0
PaCO_2_ (torr)	43 (30–46)	50 (39–73)	0.31
Base excess (mmol/L)	−28 (−30–−24)	−28 (−30–−26)	0.85
Lactate (mmol/L)	20 (19–20)	20 (18–20)	0.91
Characteristics 45 min after ROSC			
Heart rate (bpm)	213 (168–234)	190 (152–219)	0.56
Mean arterial blood pressure (mmHg)	50 (42–58)	42 (31–57)	0.41
Diastolic pressure (mmHg)	38 (27–50)	33 (22–49)	0.55
Carotid blood flow (ml/min)	26 (19–46)	29 (25–39)	0.69
Arterial pH	7.11 (6.92–7.24)	7.02 (6.93–7.19)	0.91
PaO_2_ (torr)	86 (66–101)	81 (76–89)	0.91
PaCO_2_ (torr)	34 (28–45)	37 (33–57)	0.56
Base excess (mmol/L)	−21 (−26–−14)	−20 (−23–−15)	0.73
Lactate (mmol/L)	18 (14–19)	16 (14–19)	0.73

Data are presented as median (IQR); CC, chest compression; ROSC, return of spontaneous circulation; PaO_2_, arterial oxygen partial pressure; PaCO_2_, carbon dioxide partial pressure.

### Resuscitation and primary outcome

Table 2 presents a summary of asphyxia and resuscitation outcome measures. The number of piglets that achieved ROSC was 5 (71%) and 5 (71%) with 60/min and 90/min CC rates, respectively (*p* = 1.00). The median (IQR) resuscitation time was 132 (71–600) s for CC rate of 60/min and 189 (96–600) s for CC rate of 90/min (*p* = 0.46), which includes piglets that did not achieve ROSC and received the maximum duration of resuscitation. When piglets who did not achieve ROSC were excluded, the median (IQR) time to achieve ROSC was 97 (65–149) s for CC rate of 60/min and 136 (88–395) s for CC rate of 90/min (*p* = 0.31). The number of piglets requiring epinephrine was 4 (57%) and 5 (71%) with 60/min and 90/min CC rates, respectively (*p* = 1.00), and they received 1(0–3) and 1(0–3) doses of epinephrine with CC rates of 60/min and 90/min, respectively (*p* = 0.62). Following ROSC, survival to 45 min was achieved by 5 (100%) piglets in the 60/min group and 4 (80%) piglets in the 90/min group (*p* = 1.00). The median (IQR) survival time after ROSC was 45 (45–45) min and 45 (23–45)min in the 60/min and 90/min groups, respectively (*p* = 0.69).

**Table 2:** Characteristics of asphyxia, resuscitation, and survival of asphyxiated piglets.

	60/min CC rate (*n* = 7)	90/min CC rate (*n* = 7)	*p*-value
Asphyxia time (sec)^†^	440 (310–490)	440 (280–506)	1.00
Requiring epinephrine (*n*)	4 (57%)	5 (71%)	1.00
Epinephrine doses^†^	1 (0–3)	1 (0–3)	0.62
Resuscitation time^a^ (sec)^†^	132 (71–600)	189 (96–100)	0.46
Achieving ROSC (*n*)	5 (71%)	5 (71%)	1.00
Time to achieve ROSC^b^ (sec)^†^	97 (65–149)	136 (88–395)	0.31
Survival after ROSC (% change after ROSC)	5 (100%)	4 (80%)	1.00
Survival time after ROSC (min)^†^	45 (45–45)	45 (23–45)	0.69

Data are presented as *n* (%), unless indicated ^†^median (IQR); group comparison with Mann Whitney test.

^a^ Time of all piglets resuscitated (if no ROSC, time = 600 s).

^b^ Time of piglets that only achieved ROSC.

### Hemodynamic and respiratory parameters

**Figure 2:**
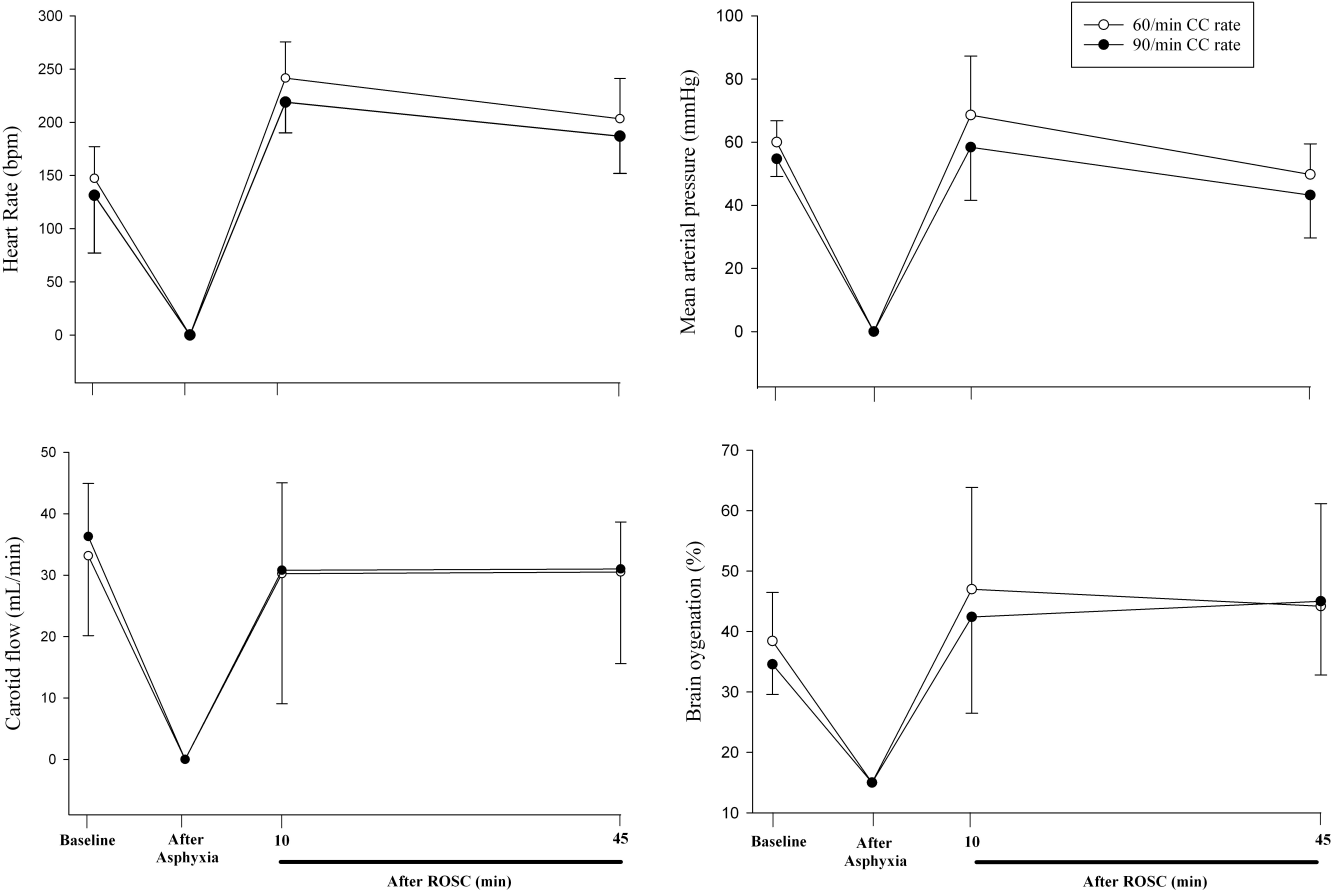
Changes in heart rate, mean arterial blood pressure, carotid blood flow, cerebral oxygenation throughout the experiment.

**Figure 3:**
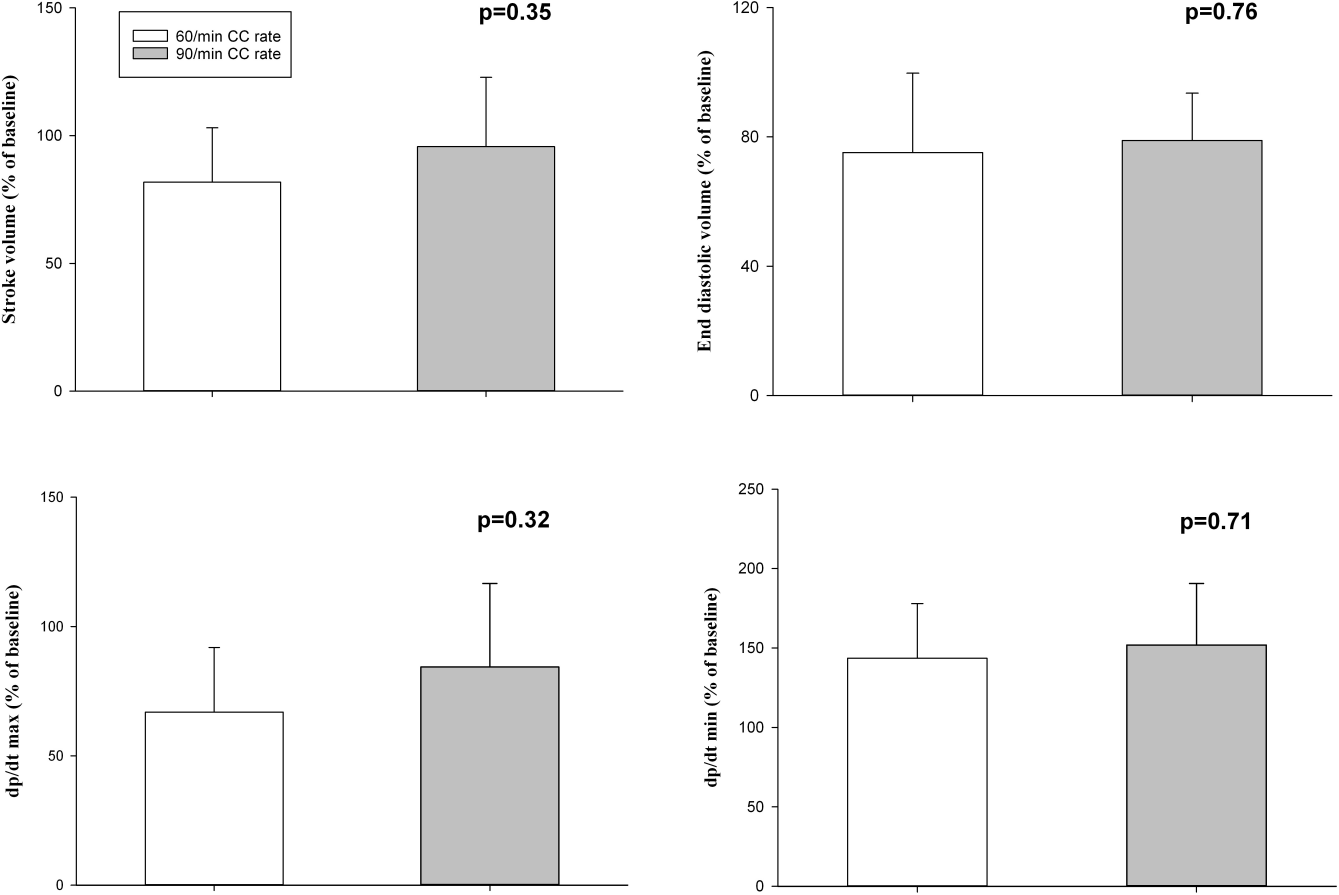
Cardiac function parameters during cardiopulmonary resuscitation.

Hemodynamic parameters at baseline and at commencement of CPR were not different (Table 1). Changes in heart rate, mean arterial blood pressure, carotid blood flow, and cerebral oxygenation throughout the experiment are presented in Figure 2. Changes in stroke volume, end-diastolic volumes, dP/dt_max_ (maximal rate of rise of left ventricular pressure), and dP/dt_min_ (minimum rate of change of ventricular pressure) during CPR are presented in Figure 3.

There was no difference in respiratory parameters between groups, which are presented in Table 3.

**Table 3:** Respiratory parameters during resuscitation.

	60/min CC rate (*n* = 7)	90/min CC rate (*n* = 7)	*p*-value
Tidal volume (ml/kg)	5.3 (0.6)	5.8 (0.9)	1.00
Minute ventilation (ml/kg/min)	436 (83)	522 (79)	0.31
Peak inspiratory flow (L/min)	4.1 (3)	3.7 (0.4)	1.00
Peak expiration flow (L/min)	−5.3 (1.9)	−5.8 (0.7)	0.46
Peak inflation pressure (cmH_2_O)	28.9 (2.2)	30.0 (1.5)	1.00
Positive end expiratory pressure (cmH_2_O)	22.1 (10.6)	29.1 (2.0)	0.33
Rate (/min)*	60 (1)	90 (1)	

Data are presented as the mean (SD).

* Rate = Ventilation and number of chest compressions, which corresponds to the number of ventilations per minute.

## Discussion

The current CoSTR for neonatal resuscitation recommends providing 90 CC and 30 inflations (=120 events/min) to optimize cardiac output and oxygen delivery (1, 2). However, the optimal CC rate during neonatal CPR remains unclear. While a mathematical study suggests that the most effective CC rate depends upon body size and weight, and that CC rates as high as 180/min might improve survival in newborn infants (21), these rates are near impossible to achieve with any manual technique during neonatal resuscitation.

In the current study, we compared CC rates of 60/min and 90/min using CC + SI. The results can be summarized as follows: (1) time to ROSC and the number of piglets achieving ROSC was not different (Table 2), (2) heart rate, arterial pressure, carotid blood flow, and cerebral blood oxygen saturation were not different (Figure 2), (3) cardiac function parameters—stroke volume, end diastolic volume, and left ventricle contractile function (dP/dt_min_ and dP/dt_max_)—were not different (Figure 3), and respiratory parameters were not different (Table 3) between groups. The lack of differences in any outcome might be of clinical significance because it indicates that rather than focusing on achieving the correct rate, healthcare providers could be taught to focus on adequate technique and compression depth instead.

In a simulated neonatal CPR manikin study, Li et al. demonstrated a greater decay in CC depth when a faster rate was used over a 10 min resuscitation period (22). Continuous CC with asynchronized ventilations was delivered at a rate of 120/min and 90/min. After 3 min, CC depth was reduced by 50% and 30% when using rates of 120/min and 90/min, respectively (22). By the end of the 10 min resuscitation period, CC depth was even further reduced by 70% and 40% of baseline values with rates of 120/min and 90/min, respectively (22). In the current study, we demonstrate that using a slower CC rate (60/min) did not adversely affect survival or cardiopulmonary outcome compared to a faster rate of 90/min. Using a slower rate may delay the onset and severity of CC depth deterioration, and could improve the quality of delivered CC. This may also improve healthcare providers’ perceived fatigue during resuscitation efforts. Indeed, we used a custom-designed CC machine (12, 13), which allowed consistent delivery of CC rates and reduced potential bias (e.g., fatigue during CC or inability to constantly achieve allocated rate). While using CC rates of 60/min might reduce fatigue, further studies are needed to assess this in more detail.

There is a lack of studies examining CC rates slower than 90/min. Solevåg et al. performed a study investigating alternating 9 CC and 3 ventilations in asphyxiated piglets with cardiac arrest, with the hypothesis that 9 CC would generate higher diastolic blood pressure during CPR than only 3 CC per series (23). However, increasing the number of CC in a row should not be at the expense of ventilation, hence the ratio of CC to ventilation was maintained at 3:1. The two approaches provided comparable mean CC per minute (54 vs. 53 for 3:1 C:V and 9:3 C:V, respectively), and the time to ROSC was also similar (median time of 150 s and 148 s for 3:1 C:V and 9:3 C:V, respectively). In addition, there were no differences in diastolic blood pressure during CC (23). Similarly, C:V ratios of 3:1 and 15:2 were compared using the same model (24). Although the 15:2 C:V ratio provided higher mean CC per minute (75 vs. 58 for 3:1), time to ROSC was similar between groups (median time of 195 s and 150 s for 15:2 and 3:1, respectively) (24).

We recently compared cardiac function [i.e., stroke volume, cardiac output, and left ventricular function (dP/dt_max_ and dP/dt_min_)] with CC rates between 60 and 180/min (25). Stroke volume and cardiac output were highest with a CC rate of 180/min, while end-diastolic volume, dP/dt_max_ or dP/dt_min_ was highest at a CC rate of 150/min. A further increase to a CC rate of 180/min did not further increase the end-diastolic volume, dP/dt_max_, or dP/dt_min_ (25). Although a slower rate may have beneficial effects on rescuer fatigue, these data suggest that CC with a rate of 150–180/min might have optimal cardiovascular performance.

During neonatal resuscitation, Patel et al. randomized asphyxiated transitioned newborn piglets to continuous CC with asynchronized ventilation with CC rates of 90/min, 100/min, and 120/min and reported similar mean times to ROSC (26). However, the piglets in the 90/min and 100/min groups had higher cerebral inflammation and brain injury than those in the 120/min group (26). Data from an adult resuscitation study in pigs may provide insight on this neuroprotective aspect of a higher CC rate. Taylor et al. showed that regularly switching CC rates between 100/min and 200/min throughout resuscitation improved hemodynamics compared to CC with a constant rate of 100/min (27). Interestingly, switching to a rate of 100/min increased coronary perfusion pressure, while switching to 200/min increased cerebral perfusion pressure. This indicates that different CC rates may provide perfusion pressures that favor the brain or the heart. A possible explanation is that higher CC rates may lead to lack of time for left ventricular and thoracic blood vessel filling, but may improve the emptying of the left ventricle and thorax of blood, thus could improve net forward blood flow. It may be that a single optimal CC rate for neonatal resuscitation does not exist, but rather a rate-variable approach that is both neuro- and cardio-favorable would be better suited.

Mathematical models describe that the maximal cardiac output during CPR depends on the fraction of cycle time available for pump filling and emptying, with pump filling being dominant (21, 28). Fitzgerald et al. compared CC rates between 20 and 140/min in 6–16 kg adult mongrel dogs and reported the maximum cardiac output would be achieved with a CC rate of 126/min (28). The physiological heart rate in neonates ranges between 120 and 160/min (29), and an increase in the CC rate might have the potential to boost artificial cardiac output compared to recommended CC rates, which are based largely on experimental work in animal models larger than neonates. The mathematical model by Babbs et al. calculated the optimal CC rate for maximum cardiac output with a CC rate of 184/min for a 3 kg newborn infant, indicating an effect of body size and weight (21).

The current CoSTR for neonatal resuscitation recommends providing 90 CC and 30 inflations (=120 events/min) (1, 2), while the optimal rate remains unknown. A mathematical model suggests rate as high as 180–210/min based on weight to optimize cardiac output (21). Several studies compared different compression to ventilation ratios (C:V) including 2:1, 4:1, 9:3 or 15:2 with 3:1 and reported no difference in time to ROSC or mortality (23, 24, 30), however, all of these studies used a CC rate of 90/min. Similar studies comparing continuous CC with asynchronized ventilation (CCaV) using either a rate of 90/min or 120/min compared to 3:1 C:V did not reduce time to ROSC or decreased mortality (26, 31, 32). However, when CC + SI was compared to 3:1 C:V with either CC rate of 90/min or 120/min, the time to ROSC and survival was improved (4, 6). Indeed, in the original study by Schmölzer et al. comparing CC + SI with a rate of 120/min to 3:1 C:V with CC rate of 90/min the time to ROSC was significantly decreased with CC + SI 38 (23–44) s vs. 143 (84–303) s with 3:1 C:V (*p* = 0.0008) and significantly more piglets survived to 4 h after resuscitation (7/8 [87.5%] with CC + SI vs. 3/8 [37.5%] with 3:1 C:V, *p* = 0.038 (4). A follow-up study by the same group compared CC rate of 90/min during CC + SI with 3:1 C:V with a CC rate of 90/min and reported that CC + SI significantly reduced the median (IQR) time of ROSC, i.e., 34 (28–156) s vs. 210 (72–300) s in the 3: 1 group (*p* = 0.048) as well as improved survival (7/8[87.5%] vs. 4//8 [50%] *p* = 0.31, which was not statistical significant. More recently Bruckner et al. compared CC + SI with CC rates of 180/min and 90/min and reported no difference in time to ROSC or survival (7). These studies suggests, that the optimal approach of chest compression in the delivery room remains unknowns an further studies are needed to identify the best approach.

### Limitations

Although CC + SI (3) is mentioned in the knowledge gap section of the neonatal resuscitation guidelines, it is currently not a recommended treatment option (1, 2). We used a piglet asphyxia model that closely simulates delivery room events, with gradual onset of severe asphyxia leading to asystole. However, our piglets have already undergone the fetal-to-neonatal transition, are sedated/anesthetized, and we use tracheostomy with a tightly sealed endotracheal tube, which does not occur in the delivery room. A strength of this study is the use of our automated CC machine, which can concisely apply different rates of CC (7, 12, 13).

## Conclusion

There was no difference in time to ROSC and survival when CC + SI was used with a CC rate of 60/min or 90/min. Respiratory and hemodynamic parameters were similar between groups. Different CC rates might affect oxygen delivery and organ perfusion and warrants further investigation.

**Take-home message:** Using a slower chest compression rate of 60/min compared to the currently recommended rate of 90/min results in similar hemodynamic and respiratory outcomes during neonatal cardiopulmonary resuscitation. Although the ideal compression rate for optimal oxygen delivery and organ perfusion warrants further investigation, healthcare providers may focus on delivering adequate compression depth and technique rather than achieving correct rate.

## Data Availability

The original contributions presented in the study are included in the article/Supplementary Material, further inquiries can be directed to the corresponding author.
